# Online Moral Conformity: how powerful is a Group of Strangers when influencing an Individual’s Moral Judgments during a video meeting?

**DOI:** 10.1007/s12144-023-04765-0

**Published:** 2023-06-01

**Authors:** Mariola Paruzel-Czachura, Dominika Wojciechowska, Dries Bostyn

**Affiliations:** 1 Institute of Psychology, University of Silesia in Katowice, Grazynskiego 53, Katowice, 40-126 Poland; 2 Penn Center for Neuroaesthetics, Goddard Laboratories, 3710 Hamilton Walk, Philadelphia, PA 1910 USA; 3 Fylde and Wyre CAMHS (Child and Adolescents Mental Health Services), NHS Lancashire and South Cumbria Foundation Trust, Preston, UK; 4 Ghent University, Ghent, Belgium

**Keywords:** Online moral conformity, Moral judgment, Utilitarianism, Moral psychology, Video meeting

## Abstract

People make moral decisions every day. When making such decisions, they may be influenced by their companions (a so-called moral conformity effect). Increasingly, people make decisions in online environments, like video meetings. In the current preregistered experiment, we studied the moral conformity effect in an online context. We applied an Asch conformity paradigm by asking participants (*N* = 120) to reply to sacrificial moral dilemmas through the online video communication tool Zoom either when sitting in a “virtual” room with strangers (confederates instructed on how to answer; experimental condition) or when sitting alone (control condition). We found that people displayed a moral conformity effect on half the dilemmas included in our study as well as in the aggregate.

**Supplementary Information:**

The online version contains supplementary material available at 10.1007/s12144-023-04765-0.

At the age of seven, a young Solomon Asch took part in the celebration of the Jewish Passover in the small Polish town where he grew up. In accordance with tradition, a cup of wine was placed at an empty spot on the table. When the young Asch enquired why, he was told it was waiting for the prophet Elijah, who visits each Jewish home on Passover and takes a sip of wine from the cup left out for him. An uncle suggested he keep a close eye on the glass and thus, a young Asch kept watching the cup to see what happened. Would the prophet take a sip of wine? That night, the hopeful boy was convinced he saw the level of wine dip just a little bit (Stout, 1996). Many years later, Asch said that this childhood experience influenced his group pressure and conformity studies.

It is hard to overstate the power of suggestion. Indeed, later in life, Asch would go on to demonstrate that even our most basic perceptions, such as the length of the line, can be influenced by the suggestions of others (Asch, 1951). In his seminal work, Asch famously asked participants to gauge the length of a series of lines in front of a group of actors, intentionally giving wrong answers. Although this task was straightforward and specifically designed to be easy, in about one-third of all trials, participants conformed to the obviously wrong answers given by the group (Asch, 1952, 1955, 1956). By debriefing his participants, Asch uncovered that most of his participants conformed because they thought they must have misinterpreted the stimuli (what Asch termed the “distortion of judgment”) or because they did not want to seem out of step with the group (a “distortion of action”). However, a small number of participants appeared to be genuinely unaware that they were giving clearly wrong answers (a “distortion of perception”). The suggestion of a couple of peers was sufficient to blind people from an obvious truth.

Asch’s work on conformity has been some of the most influential in social psychology and has withstood the test of time. Variations of Asch’s procedures have been conducted numerous times, and the findings are easily replicated (Bond, 2005; Bond & Smith, 1996). Conformity, i.e., changing one’s attitude or behavior to match a perceived social norm, really exists. Accordingly, one may wonder: if the judgments of others can influence how people judge the length of the lines and cause them to distrust their own eyes, would the power of suggestion also work in moral issues? Could it be possible for an individual to change their opinion about the moral appropriateness of killing another human?

Indeed, there is research that has confirmed that conformity effects can be found in the moral domain as well (Chituc & Sinnott-Armstrong, 2020). Most of these studies have investigated moral conformity by confronting participants with written fake responses of a group’s moral decisions and uncovered that these could be effective in changing people’s moral opinions (Aramovich et al., 2012; Bostyn & Roets, 2017; Crutchfield, 1955; Kelly et al., 2017). However, psychologists have also uncovered that real-life interaction can be critical for conformity effects (Allen, 1966; Deutsch & Gerard, 1955; Levy, 1960). Importantly, the existence of the moral conformity effect has been corroborated through studies using the Asch paradigm as well (Kundu & Cummins, 2013; Lisciandra et al., 2013).

While the aforementioned studies go a long way in demonstrating that moral conformity effects exist, the world we live in today consists of more types of interaction than when Asch first conducted his experiments. More and more, human interaction has moved from the real world to online, a shift that has only accelerated due to the COVID-19 pandemic. For instance, the video communication tool Zoom reports that its daily users rose from 10 million in December 2019 to over 200 million by the second quarter of 2020 (Yuan, 2020). Already, the two younger generations, Gen Z and Millennials, communicate more through digital communication channels than in person (Brandury, 2018). As more and more of our communication shifts online, one must wonder: does the power of suggestion also work in online places?

## The age of online video meetings

One type of digital communication that has seen incredible growth in the past few years is video communication. As a result of the COVID-19 pandemic, work-from-home mandates, and lockdowns, more and more people have turned to video communication tools to talk with family members and friends, schedule work meetings, or attend social events (Cataldo et al., 2021; Greenhalgh et al., 2020). Video communication has several advantages (Johns et al., 2021; Karl et al., 2022). It is often more convenient as people can communicate from the comfort of their own homes and offices, even with others half a world away. In contrast to text-based communication, it does not obscure body language or prosody, and it can allow users to express themselves with more nuance than when communicating through more traditional online channels. Most video communication tools are flexible, allowing users to schedule meetings on the fly with up to hundreds of participants (Houston, 2020). As a result, video communication can often be more efficient and cost-effective than in-person communication. Out of all current forms of digital communication, video communication likely mimics real-life interactions the closest.

Nevertheless, at the same time, video communication presents its own set of challenges: the quality of the communication can be hampered by a bad connection. This can cause a blurring of the image, delays, jerkiness, or out-of-sync audio. People need to cognitively correct such distortions, which require constant and sustained attention. As a result, video communication can be more tiring than traditional forms of communication (Nesher Shoshan & Wehrt, 2021; Bennet et al., 2021) and lead to an increased feeling of disconnect between the different communication partners (Bailenson, 2021; Sherman et al., 2013). This problem is exacerbated as most people cannot look each other in the eye when using video communication tools. Most video cameras are angled above or below the viewing screen. Thus, people typically appear to be looking up or down rather than directly at each other, which inhibits the formation of mutual trust (Bayliss & Tipper, 2006; Mason et al., 2005). Furthermore, several studies have shown that people tend to look more at themselves in video calls than they do at others (Devue et al., 2009). Prior research has established that face-to-face interactions can be critical for conformity effects (Allen, 1966; Deutsch & Gerard, 1955; Levy, 1960). While video communication involves face-to-face interaction, these findings suggest that the type of face-to-face interaction people have through video communication is likely to be of decreased quality. Whether this impacts the extent to which people conform is an open research question.

## Morality and conformity

The need to perceive ourselves as moral is an essential psychological trait (Prentice et al., 2019). Moreover, people strive to be more moral (Sun & Goodwin, 2020), and morality is central to their sense of self and feelings of individual identity (Aquino & Reed, 2002; Blasi, 2005; Paruzel-Czachura & Blukacz, 2021). More than any other kind of change (e.g., a change to one’s physical appearance or cognitive abilities), a dramatic change to someone’s moral preference can cause people to proclaim that someone is no longer their true self (Strohminger & Nichols, 2014). As such, one might assume that people are unlikely to change their moral judgments and, thus, unlikely to conform to moral matters. After all, spoken moral judgments are some of the strongest signals of our moral identity. Furthermore, although some studies are showing how easily we may change others’ moral judgments, most studies confirmed that philosophical intuitions are surprisingly stable (see the review: Knobe, 2022), nor do people change their moral judgments when their emotions are manipulated (see the meta-analysis: Landy & Goodwin, 2015) or when they are drunk (Paruzel-Czachura et al., 2021 as an unsuccessful replication of Duke & Bègue, 2015). Nevertheless, one consistent factor that does seem to impact individuals’ thoughts about right or wrong is group norms (Chituc & Sinnott-Armstrong, 2020).

Studies on moral conformity are centered on the question of whether a group can influence an individual’s moral judgment. Despite its theoretical and practical relevance, there is surprisingly little research on this topic, and past research has uncovered some contradictory results. On the one hand, research showed that written statistics about group preference (e.g., that 70% of people consider a specific option to be moral) could be enough to change people’s moral judgments (Aramovich et al., 2012; Bostyn & Roets, 2017; Crutchfield, 1955; Kelly et al., 2017). On the other hand, mixed results were found in studies based on Asch’s paradigm. While both Kundu and Cummins (2013), as well as Lisciandra et al. (2013), found a conformity effect when asking participants, along with a group of confederates, to respond verbally to moral dilemmas the latter group of authors did not find a conformity effect when participants only read the responses of the confederates on a computer screen and were asked to respond digitally. Interestingly, no prior work has tested whether moral conformity effects persist in videoconference settings. Because of the popularity of online interactions nowadays, studying to what extent moral conformity effects exist in such settings would be valuable.

## The current research

We aimed to investigate whether the moral conformity effect emerges when participants are asked to respond to a series of moral dilemmas (see Table 1) administered through video conferencing software. We applied an Asch conformity paradigm in an online context by asking participants (and confederates) to reply to moral dilemmas through the online video communication tool Zoom.

Past research showed that the more complex the question posed to participants is, the more they tend to demonstrate conformity effects (for a review: Sunstein, 2019). As moral dilemmas require participants to perform the complex task of weighing conflicting moral values, we suspected the conformity would be even higher than in classical Asch’s studies (in which individuals conformed in one-third of all trials). Moreover, responses to moral dilemmas are perhaps more subjective than judgments about the length of lines. Accordingly, we expected to observe an online moral conformity effect.

Additionally, we wanted to test if participants would be more or less conformist depending on the type of moral dilemmas they are confronted with: personal or impersonal dilemmas. Personal dilemmas are those where all of the following conditions are met: anticipated harm done by sacrificing someone leads to serious harm, damage to health, or even death, and results directly from the hero’s actions dilemma, not from “shifting” an already existing threat to someone else, and if the harm resulting from the sacrifice affects a specific person or member(s) specific group (Greene et al., 2001). The criterion for using “personal power” Greene and colleagues (2009) define it as an action that directly uses force muscles (e.g., pushing someone off with your own hands) and not indirectly (e.g., by shooting from a gun). Greene’s dilemmas (Greene et al., 2008) gained great popularity among researchers dealing with morality and were used, among others, in the studies mentioned above by Kundu and Cummins (2013) or Bostyn and Roets (2016). We preregistered the hypothesis that participants would be more conformists in impersonal moral dilemmas than personal ones because past studies showed that it was much easier to participants do act in impersonal dilemmas (e.g., move the switch) than personal ones (e.g., push the man from the bridge) (Bago et al., 2022). We assumed it would also be easier for participants to follow actors’ acting in impersonal dilemmas than personal dilemmas.

The current study was preregistered at https://osf.io/832eq. All data, materials, and analysis scripts are available at https://osf.io/gdb52/. All analyses were conducted in R (R Core Team, 2015). The study was approved by the Ethics Committee of University of Silesia in Katowice. We report how we determined our sample size, all data exclusions, all manipulations, and all measures in the study (Simmons et al., 2011).

## Method

### Participants

A power analysis suggested that a sample of *n* = 50 per experimental condition would have at least 80% power to detect a shift of 15% in response tendencies (Faul et al., 2009). Accordingly, we strived for a minimal sample size of 100 participants, while aiming for a slightly higher sample size of 120 participants. We planned to stop the data collection the day we would achieve *N* = 120. If more participants would sign-up on the same day, we planned to study all of these participants. All future appointments after the day of completion were canceled.

A sample of *N* = 120 adults (71 females, *M*_age_: 26.18 years, *SD* = 9.05) volunteered for the online experiment. Participants were recruited through advertisements on local websites, including Facebook and Instagram, and in local newspapers in spring 2021. Participants took part in the study voluntarily, with no monetary compensation. Participants were required to be at least 18 years old and were not allowed to be students in psychology or graduate students in psychology. Participants were predominantly catholic (*N* = 87, 4 others, 29 atheists). Among believers, religious practice was on an average level of *M* = 2.74 (*SD* = 2.28) on a scale of 0 (*not practicing at all*) – 7 (*very religious*). Detailed statistics about participants’ education and employment status are in Supplementary Materials. No other data was collected, and data from all participants were analyzed.

### Procedure and materials

Participants completed an online survey confirming the above conditions and made an appointment. They were asked about their age, sex, education level, occupational status, religion, and practicing religion, given an anonymous code for future contact with the researcher, and were asked to provide an anonymous e-mail address they preferred to use to receive the link to the study.

**Fig. 1:**
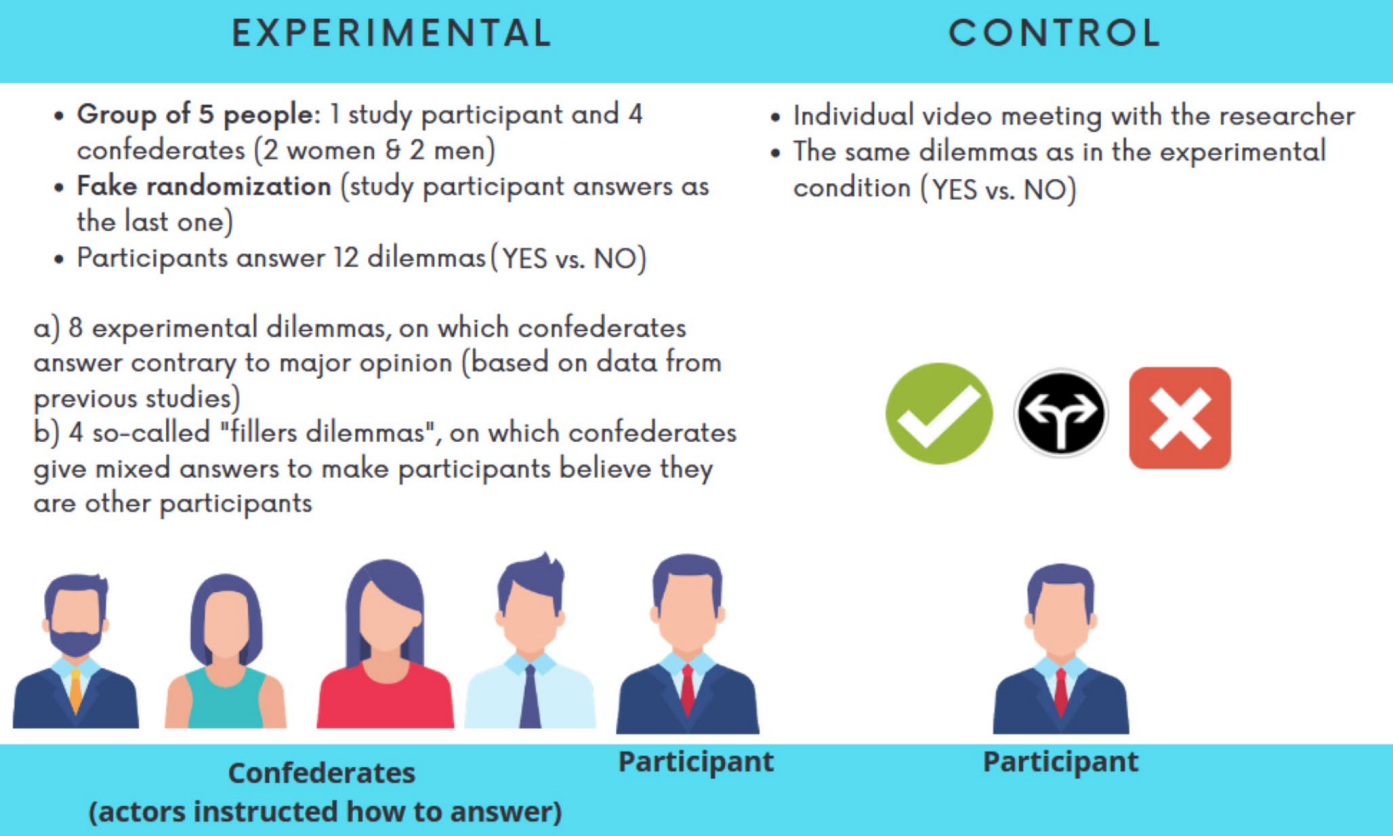
Overview of the Study Procedure

The study was run through the Zoom platform (and participants joined Zoom using their unique anonymous code). Every experimental session was run as a video meeting that was recorded. Before the Zoom meeting, participants had to sign an online agreement to participate in the study according to the ethical requirements. Figure 1 presents an overview of the procedure.

Participants were randomly assigned to either the experimental or the control group conditions. In the experimental group, they answered orally on the moral dilemmas in group settings, but other participants were actors – confederates (two women and two men of a similar age) who were previously instructed how to answer. Each session started with the following instruction: “You will always answer in the same queue. I am randomizing your queue now (the researcher pretends to use some randomizer). Ok. So, CODE1 (female confederate), you will answer as the first, CODE2 (male confederate), you are the second, CODE3 (female confederate), you are the third, CODE4 (male confederate), you are fourth, and CODE5 (study participant) you are the fifth”. In the control condition, participants took part in the study alone, without any confederates.

The list of dilemmas and actors’ answers for the experimental condition is available in Table 1. In each group (experimental and control), participants saw 12 randomized dilemmas on the screen (8 experimental and 4 so-called fillers). Participants were asked to decide if the proposed solution to the dilemma was acceptable or unacceptable (*yes* vs. *no*).

This kind of procedure could make our participants suspect the true aim of the study. So, we used two remedies to improve our procedure and make our participants believe the confederates were also participants like them. First, we did not invite participants who had received at least one psychology course before in their life. Second, similar to the procedure Asch used, we included filler trials, i.e., additional dilemmas on which confederates answered differently (gave mixed answers) or similarly (when the answer to the dilemma was obvious, i.e., “yes, it is wrong to kill an innocent person to save some sculpture”). As was clear from the debriefing we conducted after the study, none of the participants realized that we aimed to study conformity.

The dilemmas used were taken from prior research, and actors’ answers were based on the responses given to these dilemmas in said research (Gawronski et al., 2017; Greene et al., 2008; Körner et al., 2020). For example, for the set of 8 experimental dilemmas, when most people in a particular dilemma answered “yes” in previous studies, we asked all actors to say “no”. For the set of 4 fillers, confederates answered differently on three of the fillers to make the study more reliable (e.g., the first actor: “yes”, the second actor: “no”, the third actor: “no”, the fourth actor: “yes”). On one of the fillers, all confederates answered the same way as participants did in previous studies. The entire procedure with detailed sentences used by the experimenter is available at OSF: https://osf.io/832eq.

**Table 1:** The List of Dilemmas and Actors’ Answers in the Experimental Condition

Type of Dilemma	The Dilemma	Actor’s Answers (1st actor, 2nd actor, 3rd actor, 4th actor)
**[filler]**	“Grandson” (Greene et al., 2008). A young boy is visiting his grandmother for the weekend. Usually she gives him a gift of a few dollars when he arrives, but this time she doesn’t. He asks her why not and she says something about how he doesn’t write her as many letters as he used to. Angered by this, the boy decides to play a trick on her. While his grandmother is busy cooking he removes a handful of pills from the medicine cabinet and puts them in his grandmother’s teapot. Later that night, she makes her tea, and the dissolved pills make her sick. In the morning she is dead. Was it appropriate for this boy to put pills in his grandmother’s teapot in order to play a trick on her?	All NO
**[filler]**	“Speedboat” (Greene et al., 2008). While on vacation on a remote island, you are fishing from a seaside dock. You observe a group of tourists board a small boat and set sail for a nearby island. Soon after their departure you hear over the radio that there is a violent storm brewing, a storm that is sure to intercept them. The only way that you can ensure their safety is to warn them by borrowing a nearby speedboat. The speedboat belongs to a miserly tycoon who would not take kindly to your borrowing his property. Is it appropriate for you to borrow the speedboat in order to warn the tourists about the storm?	YES, NO, YES, NO
**Impersonal**	“Transplant” (Gawronski et al., 2017). You are a surgeon in a small hospital. One day, your hospital receives five badly hurt patients from a car accident. These patients all need organ transplants or they will have serious health problems for the rest of their lives. You have no spare organs, but there is a patient who has been in a coma for several weeks and it seems unlikely that he will wake up again. One of your co-workers plans to terminate his life support and take his organs for the five accident victims, so that they won’t suffer from health problems. You could stop your co-worker by informing the director of the hospital. Is it acceptable in this case to stop your co-worker from terminating the patient’s life support to take his organs?	All NO
**Impersonal**	“Tyrant killing” (Gawronski et al., 2017). You are head chef of a restaurant in a South American country governed by a controversial president. Since he came to power, he has been trying to suppress any opposition. Many people who dared to speak up against him suddenly “disappeared” and later their dead bodies were found in mass graves. The president is visiting the restaurant in which you work. You know he is highly allergic to peanuts, and you notice that a waiter accidentally mixed up his peanut-free dish with one that contains a small amount of peanut oil. Although the amount is too small to be noticeable, it would be enough to kill him and provide a chance for a new political era in your country. Is it acceptable in this case to prevent the waiter from serving the peanut dish?	All YES
**Personal**	“Hard times” (Greene et al., 2008). You are the head of a poor household in a developing country. Your crops have failed for the second year in a row, and it appears that you have no way to feed your family. Your sons, ages eight and ten, are too young to go off to the city where there jobs, but your daughter could fare better. You know a man from your village who lives in the city and who makes sexually explicit films featuring small children such as your daughter. He tells you that in one year of working in his studio your daughter could earn enough money to keep your family fed for several growing seasons. Is it appropriate for you to employ your daughter in the child pornography industry in order to feed your family?	All YES
**[filler]**	“Sculpture” (Greene et al., 2008). You are visiting the sculpture garden of a wealthy art collector. The garden overlooks a valley containing a set of train tracks. A railway workman is working on the tracks, and an empty runaway trolley is heading down the tracks toward the workman. The only way to save the workman’s life is to push one of the art collector’s prized sculptures down into the valley so that it will roll onto the tracks and block the trolley’s passage. Doing this will destroy the sculpture. Is it appropriate for you to destroy the sculpture in order to save this workman’s life?	YES, YES, NO, YES
**Impersonal**	“Torture” (Gawronski et al., 2017). You are a member of a special police department who is trained to obtain information in particularly difficult cases. You are dealing with a case involving a male adult who is accused of having stolen several paintings. You don’t know where he is hiding the paintings, and he refuses to tell you where they are. The paintings will likely be shipped to a different country if they are not found within the next 24 h. You have tried every legal interrogation technique, but none of them were successful. To get information on where the paintings are, your partner starts using illegal techniques that are deemed torture, and you consider stopping him by reporting him to your supervisor. Is it acceptable in this case to stop your partner from using illegal interrogation techniques?	All NO
**Personal**	“Submarine” (Greene et al., 2008). You are the captain of a military submarine traveling underneath a large iceberg. An onboard explosion has caused you to lose most of your oxygen supply and has injured one of your crew who is quickly losing blood. The injured crew member is going to die from his wounds no matter what happens. The remaining oxygen is not sufficient for the entire crew to make it to the surface. The only way to save the other crew members is to shoot dead the injured crew member so that there will be just enough oxygen for the rest of the crew to survive. Is it appropriate for you to kill the fatally injured crew member in order to save the lives of the remaining crew members?	All NO
**Personal**	“Modified bomb” (Greene et al., 2008). You are negotiating with a powerful and determined terrorist who is about to set off a bomb in a crowded area. Your one advantage is that you have his teenage son in your custody. There is only one thing that you can do to stop him from detonating his bomb, which will kill thousands of people if detonated. To stop him, you must contact him over the satellite hook-up that he has established and, in front of the camera, break one of his son’s arms and then threaten to break the other one if he does not give himself up. Is it appropriate for you to break the terrorist’s son’s arm in order to prevent the terrorist from killing thousands of people with his bomb?	All NO
**[filler]**	“Architect” (Greene et al., 2008). You are a young architect visiting one of your construction sites with your boss. Your boss is a despicable individual who makes everyone around him miserable including you. It occurs to you that if you were to push him off of the building you are inspecting he would fall to his death and everyone would think it was an accident. Is it appropriate for you to push your boss off of the building in order to get him out of your life?	YES, NO, YES, NO
**Impersonal**	“Dialysis” (Gawronski et al., 2017). You are the director of a dialysis clinic for patients with severe kidney problems. You are dealing with the case of a female patient with a kidney dysfunction who got food poisoning from a meal at a local restaurant. To remove the toxins from her blood, she requires an uninterrupted 24-hour dialysis session instead of the typical 4-hour session required by your other patients. If the woman does not complete the 24-hour treatment, the toxins in her blood will cause irreversible damage to her organs from which she will die. The woman has received treatment for 30 min, but you have two other patients who need their regular 4-hours treatments and you have only one dialysis machine available right now. If these patients do not receive their regular treatments within 24 h, they may experience serious nausea for the next two days. Is it acceptable in this case to stop the female patient’s 24-hour dialysis session?	All YES
**Personal**	“Transplant” (Greene et al., 2008). You are a doctor. You have five patients, each of whom is about to die due to a failing organ of some kind. You have another patient who is healthy. The only way that you can save the lives of the first five patients is to transplant five of this young man’s organs (against his will) into the bodies of the other five patients. If you do this, the young man will die, but the other five patients will live. Is it appropriate for you to perform this transplant in order to save five of your patients?	All YES

## Results

As per our preregistration, we first evaluated whether we could uncover an effect of online conformity condition on each dilemma using two-sample proportion tests. As we did not expect to find an effect in one specific direction on filler dilemmas, we used two-sided tests to analyze these dilemmas. In contrast, per our preregistration, we used one-sided tests in the direction of the expected conformity effect to analyze the responses to the experimental dilemmas. Table 2 summarizes these results.

**Table 2:** The proportion of Utilitarian Responses in the Control and Experimental Conditions for Each Dilemma and the Relevant Chi-Squared Two Sample Proportion Test. Note That Two-sided Tests Were Used for Filler Dilemmas and One-Sided Tests for all Experimental Dilemmas

	Proportion Uti (Control)	Proportion Uti (Experimental)	χ^*2*^	*p*
Filler Dilemmas:				
Grandson	0.00	0.02	0	1
Speedboat	0.90	0.92	0	1
Sculpture	0.98	0.95	0.26	0.611
Architect	0.05	0.03	0	1
Deontological Pressure:				
Transplant Impersonal	0.70	0.60	0.92	0.169
Torture Impersonal	0.77	0.63	1.94	0.082
Submarine Personal	0.68	0.57	1.28	0.129
Bomb Personal	0.78	0.62	3.21	0.037
Utilitarian Pressure:				
Tyrant Impersonal	0.60	0.68	0.58	0.223
Dialysis Impersonal	0.05	0.23	6.85	0.004
Hard Times Personal	0.00	0.17	8.84	0.001
Transplant Personal	0.00	0.15	7.69	0.003

As could be expected, we found no evidence for conformity effects on each of the four filler dilemmas. In contrast, such evidence did emerge for the experimental dilemmas. While we did not find a statistically significant difference in each of these dilemmas, such evidence did emerge in half of these dilemmas. Furthermore, in all cases, numerically all mean differences were in the expected direction.

While we had not preregistered this, we wanted to test whether a moral conformity effect would emerge on the aggregate across dilemmas. We thus conducted a mixed-model analysis to corroborate this analysis. Additionally, we used this approach to investigate whether this conformity effect might be moderated by the personal or impersonal nature of the dilemmas. First, we coded the direction of the conformity pressure for each dilemma. We then ran a generalized logistic mixed model with participants’ responses as the dependent measure and condition (control vs. experimental), direction of conformity pressure (utilitarian vs. deontological pressure), and the type of dilemma (personal vs. impersonal) as well as the interactions between these variables as predictors. While we initially included a random intercept to model the repeated nature of the dilemma responses, including this random intercept caused the model to fail convergence as the variance of the random intercept was estimated to be zero. This implies that once we account for the predictors included in the model, the variability at the subject level was not significantly larger than the random variability one would expect in the response. Accordingly, we dropped this random intercept from the model (as per Bates et al., 2018).

A subsequent analysis based on the same model without any random effects revealed a significant main effect of the direction of conformity pressure, *z* = -6.14, *p* < .001, *OR* = 0.18, and an interaction effect between the direction of conformity pressure and experimental condition, *z* = 2.85, *p* = .004, *OR* = 3.00. None of the other effects reached significance (all |*z*| ≤ 0.27, all *p* ≥ .791, all *OR* ≤ 1.11), although the main effect of the condition was just shy of statistical significance, z = -1.92, *p* = .055, *OR* = 1.71. Importantly, the parameter estimates and the statistical significance of effects estimated with the model that did not include random intercepts are essentially the same as those estimated through the model that did include the random intercepts.

Taken together, these findings confirm that the moral conformity effect persists in video interactions. While our results do not suggest that conformity effects differ for different types of dilemmas (contra our preregistered expectations), these results replicate earlier results (conducted among face-to-face non-online meetings) demonstrating conformity effects on moral judgment (Kundu & Cummins, 2013; Lisciandra et al., 2013).

## Discussion

Social conformity is a well-known phenomenon (Asch, 1951, 1952, 1955, 1956; Sunstein, 2019). Moreover, past research has demonstrated that conformity effects occur for moral issues as well (Aramovich et al., 2012; Bostyn & Roets, 2017; Crutchfield, 1955; Kelly et al., 2017; Kundu & Cummins, 2013; Lisciandra et al., 2013). However, to what extent moral conformity occurs when people interact in digital spaces, such as over video conferencing software, has not yet been investigated.

We conducted an adequately powered experimental study to determine if the effect of online moral conformity exists. Two study conditions were used: an experimental one in which study participants were answering along with a group of confederates and a control condition in which study participants answered individually. In both conditions, participants were invited to a video meeting and asked to respond orally to a set of moral dilemmas with their cameras turned on. All questions and study conditions were the same, apart from the presence of other people in the experimental condition. In the experimental condition, importantly, the experimenter pretended that all people were study participants, but in fact, only the last person was an actual study participant, and all four other participants were confederates who were trained to answer in a specific manner. Confederates answered contrary to what most people had decided in past studies (Gawronski et al., 2017; Greene et al., 2008; Körner et al., 2020). We found an effect of online moral conformity on half of the dilemmas included in our study as well as in aggregate.

Contrary to our hypothesis, we did not observe differences between personal and impersonal dilemmas. This might be related to the fact that the proposed distinction of Greene et al. (2001) may not matter in studying conformity but also to the fact that sometimes it is hard to make a clear distinction between personal and impersonal dilemmas. In some cases, using personal force may be an easy rule to follow (e.g., cut a hand vs. report some issue to a director), but in others, it may be more problematic to understand why one dilemma is personal or not. For example, in the “Tyrant killing dilemma,” in which we asked if it is acceptable to prevent the waiter from serving the peanut dish, we cannot be sure if this prevention means just saying something or pushing the waiter. It is possible that some participants may see it as a different story when imagining this dilemma. That is why we recommend using in future studies more clear distinctions to understand better how moral conformity may work depending on different types of dilemmas.

Our study is not free from limitations. First, we tested only one experimental condition: one that included, i.e., four confederates and one type of moral issue (i.e., sacrificial moral dilemmas). There is a possibility that different study conditions would bring different results as conformity effects are likely to differ depending on the number of confederates or the type of moral issue under investigation. For example, we did not test how the discussion in the group settings could impact moral conformity, and past studies showed that when participants had an opportunity to interact and discuss the best moral solution, their judgments shifted to be more utilitarian than when they made individual moral judgments (Keshmirian et al., 2022). We also investigated whether people display a moral conformity effect using a single type of dilemma, i.e., sacrificial. It is an open question whether people would display a conformity effect when confronted with dilemmas that touch on their basic moral values (Bostyn et al., 2018). On the one hand, we believe that, indeed, one could expect similar effects to other types of dilemmas, mostly because we do not see any reason why sacrificial dilemmas would be atypical in this regard. On the other hand, we do not have any evidence to support this claim. For instance, we may ask: would people change their minds about abortion (Jelen & Wilcox, 2003) after listening to the actors declare the opposite view? Perhaps responses to those kinds of dilemmas would be more resistant to group pressure. We need more studies to clarify this issue. Finally, Asch (1956) found that group size influenced whether subjects conformed. The bigger the majority group (number of confederates), the more people conformed. We did not manipulate group size and cannot say whether we would observe a higher level of moral conformity if the number of confederates was higher than in the current study.

Additionally, we did not study whether conformity effects are moderated by individual differences. For instance, we already know that people are less likely to conform if they have higher social status, receive individual profits (like money), or are highly confident about their views. However, they are more likely to conform if the task is difficult or they feel frightened (see the recent review: Sunstein, 2019). Do the same conditions matter for online moral conformity?

Moreover, there is also a possibility that different results would be obtained in non-WEIRD samples (that are White, Educated, Industrialized, Rich, and Democratic) (Henrich et al., 2010), as some research has suggested different patterns of moral judgments in non-WEIRD samples (Sorokowski et al., 2020; Turpin et al., 2021). We need replications of online moral conformity in more culturally diverse samples, controlling for used language (Białek et al., 2019).

Next, we did not focus on social media messages, which could be relevant for online moral conformity. We do not know how the present findings may relate to moral messages posted on text-based online social networks. However, we may expect moral conformity effects to occur in online social networks, as some past research showed that even written information (i.e., about how many percent of people choose some solution) has a powerful impact also in moral issues (Bostyn & Roets, 2016). The evidence suggests that people are more attentive and more likely to share and spread moral content in online interactions (Brady et al., 2020). Especially when that content triggers moral outrage. The combination of moral conformity effects and an incentive to spread moralized messages could make online environments especially sensitive to group-think and polarization. Studies on these issues would be especially interesting.

Additionally, we did not test the reasons behind the participant’s decisions. We know that conformity effects may arise for different reasons, such as distortions of judgment, distortions of action, or distortions of perceptions. It is possible that participants in the current study may have conformed because they had only a weak preference and, upon observing consensus on a moral choice, believed that this was indeed the most moral course of action. Alternatively, participants could feel that the consensus choice was not the most moral course of action but endorsed it due to group pressure. Future studies should consider this and try to understand the reasons behind online moral conformity effects. They could also measure participants’ moral judgments before the pressure condition to make sure that group really changed participants’ moral judgments.

Lastly, our sample was mainly Catholic (i.e., 87 religious people, 4 others, and 29 atheists). There is the possibility that religiosity and religion may impact our results, and our results should be interpreted in light of this sample characteristic. If religion impacts the extent to which people display online conformity effects, the relationship will likely be complex. On the one hand, religious people could be more conformist as they are obviously part of a group and might care more about the moral norms within their groups (e.g., religious people care more about binding moral foundations – including authority and loyalty) (Saroglou & Craninx, 2021). On the other hand, this might be true only for values that are relevant to the specific religion, and in fact, religion might “protect” people from conformity effects if the views pushed through conformity effects are contrary to the religious values.

In any case, the findings we have uncovered have practical importance. Our research demonstrates that the moral conformity effect persists when people communicate online through video communication tools. With more and more communication being mediated by such software, it is essential that people are aware of these dynamics.

## Conclusions

Our study showed that people follow other people’s judgments when deciding about right or wrong during online video meetings. In other words, we showed that some individuals might have problems standing against a group even if that group says something that goes against their moral values. Therefore, online moral conformity may be a dangerous “weapon” in the modern world, as group preferences can shape an individual’s moral opinion. Future work should explore both boundary conditions and possible moderation effects and investigate the extent to which online moral conformity effects differ from those found in real-life interactions.

## Electronic supplementary material

Below is the link to the electronic supplementary material.

Supplementary Material 1

## Declarations

### Open Practices

The preregistration, data, analysis codes, and study materials are available at https://osf.io/gdb52/.
