# Supplementary material for: Online Moral Conformity: how powerful is a Group of Strangers when influencing an Individual’s Moral Judgments during a video meeting?
Source: Curr Psychol. 2023 Jun 1:1–11. Online ahead of print. doi: 10.1007/s12144-023-04765-0 (PMC10233534; doi:10.1007/s12144-023-04765-0)
Supplement: Supplementary file 1 — Supplementary Material 1 [file 12144_2023_4765_MOESM1_ESM.docx]

Data about participants’ education was also obtained: 5% of the participants graduated from Junior High School, 18,3% graduated from High School, 5,8% graduated from Junior College, 33,3% of the participants were studying at a university, 25% had Bachelor degree, 11,7% Master Degree and one (0,8%) of the participants had a Doctorate. We also asked participants about their employment status. The majority of them, 35,8% (*n* = 43), were current students, 24,2% (*n* = 29) were working on a permanent contract, 6,7% (*n* = 8) were working on a casual contract, 6,7% (*n* = 8) were having their own business, and 0,8% (*n* = 1) were unemployed or retired, 0,8% (*n* = 1) at the time of the study. 12,5% (*n* = 15) of the participants were studying and working on a casual contract, 8,3% (*n* = 10) were studying and working on a permanent contract, 2,5% (*n* = 3) were studying and having their own business, 0,8% (*n* = 1) were working on a permanent contract and having own business and another 0,8% (*n* = 1) were working on a permanent contract, casual contract and having own business.
